# Biomarkers and Cellular Biology in Perioperative Medicine

**DOI:** 10.3390/cells11071147

**Published:** 2022-03-29

**Authors:** Jan Larmann, Markus M. Luedi

**Affiliations:** 1 Department of Anaesthesiology, Heidelberg University Hospital, 69120 Heidelberg, Germany; 2 Department of Anaesthesiology and Pain Medicine, Inselspital, Bern University Hospital, University of Bern, 3010 Bern, Switzerland

**Keywords:** perioperative medicine, metabolics, acute phase proteins, inflammation, trauma, anesthesiology, cardiopulmonary bypass, perioperative immunosuppression

Surgical procedures alter tissue integrity; are associated with pain and activation of the sympathetic nervous system; and sometimes, cause exposure to foreign materials used during the surgery or implanted perioperatively. Those factors have been demonstrated to induce a number of inflammatory reactions [1,2,3]. Depending on the magnitude of such perioperative trauma, the molecular and cellular impacts on homeostasis can be tremendous and can follow a bi-phasic course with perioperative inflammatory surge as well as postoperative injury-associated immunosuppression, which occur in a significant number of patients and lead to adverse outcome [2,4,5,6]. The use of cardiopulmonary bypass (CPB), for example, may aggravate perioperative immunosuppression. In addition, immunologically active drugs such as dexamethasone that are widely used to prevent perioperative nausea may trigger deleterious cellular response in patients undergoing tumor surgery [7,8]. While biomarkers and cellular biology are intensively investigated in some fields such as oncology, as of today, biomarkers and cellular responses are generally neither specifically monitored nor well understood in perioperative medicine [9].

Cardiovascular risk stratification has become an essential dimension in the standardization of clinical pathways and has significantly improved the overall quality of care [10]. However, individual treatments cannot be standardized, and the integration of individual genetic and multi-omics information bears an enormous potential to further improve the quality of perioperative outcomes [11]. In addition, integrating biomarkers, cellular biology, and clinical data offers tremendous possibilities in our journey toward comprehensive perioperative organ protection [12] and improvement of traditional risk prediction models [4,5,6,13]. As an extra level of complexity, some biomarkers, such as lipoprotein(a), can predict risk with high sensitivity and specificity for some outcomes, but the same markers’ predictive power fades in other endpoints [14,15].

This Special Issue aims to provide insights in current developments and mechanistic discoveries regarding the impact of surgical and interventional procedures on organ systems and the potential of specifically monitoring biomarkers and cellular responses in perioperative medicine.
